# Fabrication and Characterization of Olive Leaf Polyphenols Loaded Sweet Almond Gum/Gelatin Electrospun Nanofiber

**DOI:** 10.1002/fsn3.70335

**Published:** 2025-07-14

**Authors:** Shiva Geravand, Sediqeh Soleimanifard, Mohammad Amin Miri, Mahmoud Tavakoli, Atefe Rezaei

**Affiliations:** ^1^ Department of Food Science and Technology University of Zabol Zabol Iran; ^2^ School of Life Sciences, Plant Proteins and Nutrition Technical University of Munich Freising Germany

**Keywords:** electrospinning, gelatin, olive leaf, phenolic compounds, sweet almond gum

## Abstract

Olive leaf is a rich source of phenolic compounds that have antioxidant and antimicrobial properties. However, easy degradation against environmental stresses, low bioavailability, and the bitter taste of phenolic compounds are the main limitations of its use. This research was done with the aim to encapsulate olive leaf polyphenols in electrospun nanofibers of almond gum/gelatin. For this purpose, after extraction, polyphenols were loaded in different concentrations (0%, 5%, 10%, and 20%) in sweet almond gum/gelatin electrospun nanofibers. Characteristics of electrospun nanofibers of almond gum/gelatin/olive leaf polyphenol were determined by infrared spectroscopy (FTIR), X‐ray diffraction spectroscopy (XRD), scanning electron microscopy (SEM), atomic force microscopy (AFM), and thermogravimetric analysis (TGA). Moreover, the rheological properties of the electrospun solutions were investigated. Results showed that the diameter of electrospun nanofibers increased with an increase in olive leaf polyphenol concentration. AFM results showed that the morphology of the nanofibers was rod‐shaped and without disorder. FTIR and XRD data indicated that the polyphenols were effectively loaded within the carriers, and interactions were occurring between gelatin and almond gum. TGA results showed that polyphenol degradation was done in the second step. The rheometric results showed that almond gum/gelatin/polyphenol solutions behaved like Newtonian fluids and the viscosity increased with the increase in polyphenol concentration. The release rate was high in the initial times and gradually decreased with the increase of the process time. This research showed that electrospun nanofibers of sweet almond gum and gelatin loaded with olive leaf polyphenol can be used in food and medicine.

## Graphical abstract

This research was done with the aim of encapsulating olive leaf polyphenol in electrospun nano fibers of almond gum/gelatin. For this purpose, after extraction, polyphenol was loaded in different concentrations (0%, 5%, 10%, and 20%) in almond gum/gelatin electrospun nanofibers. This research showed that electrospun nanofibers of sweet almond gum and gelatin loaded with olive leaf polyphenol can be used in food and medicine.

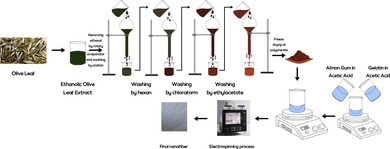

## 1 Introduction

Olive leaf is a by‐product of pruning olive trees and is a cheap and rich source of bioactive substances, especially phenolic compounds such as oleuropein, verbascoside, ligstroside, tyrosol, and hydroxytyrosol. Therefore, it has antioxidant and anti‐inflammatory properties, reduces blood pressure and blood sugar, treats chronic wounds, and is responsible for the organoleptic properties of food that can be used as a food and pharmaceutical supplement (Rahmanian et al. 2015; Xu et al. 2018).

Despite the many benefits of polyphenols, their use also entails challenges such as bitter taste, instability against oxygen, light, moisture, and heat (Khoshnoudi‐Nia et al. 2020) that limit their applications. One of the most efficient methods that have been proposed to minimize these challenges is encapsulation. Encapsulation is a process that entraps one substance into another substance, the wall material, producing particles at the nanometer (nanoencapsulation), micrometer (microencapsulation), or millimeter scale by many encapsulation techniques (Burgain et al. 2011; Chen and Chen 2007) which improves their bioavailability, controlled release, biological activity, targeting, shelf life, and masking the unpleasant taste (Popović et al. 2019).

Literature showed that nanofibers containing polyphenol are good candidates for wound dressing or medical textile applications, tissue engineering, and nano delivery systems in pharmaceuticals due to their antibacterial, antifungal, anti‐inflammatory, antiviral, and antioxidant properties (Basal et al. 2016; Bayraktar 2018; de la Ossa et al. 2021, 2022; Soleimanifar et al. 2020). Among the nanofiber production methods, electrospinning is one of the most efficient and simple methods (Kriegel et al. 2008). The process of electrospinning involves utilizing electrostatic forces to transform a pendant droplet of polymer solution into a fine fiber, which subsequently deposits onto the nearest grounded collector (Ghorani et al. 2016).

The electrospinning process allows for the use of a diverse range of polymers as wall materials for encapsulating bioactive agents. Growing concerns among researchers regarding the potential risks and health effects associated with the use of synthetic polymers have prompted greater attention toward the utilization of biopolymers (Stijnman et al. 2011; Wongsasulak et al. 2010). Biocompatible, degradable, and edible polymers, which are categorized as natural polymers, have gained significant attention in various fields such as bioengineering, pharmaceuticals, and food industries (Schiffman and Schauer 2008; Wongsasulak et al. 2010). Among the various biopolymers that can be used for electrospinning, special attention has been paid to proteins (Kriegel et al. 2008).

Recently, there has been interest in utilizing mixed biopolymers for the fabrication of nanofibers through the electrospinning technique (Aman Mohammadi et al. 2019; Schiffman and Schauer 2008).

Gelatin is a semitransparent, colorless, brittle, and tasteless biodegradable, multifunctional biopolymer with amphoteric properties, safety, water solubility, emulsification, and low cost that is obtained from the collagen of animal bones. It is composed of 19 amino acids and thus can be hydrolyzed by several proteolytic enzymes to produce its constituent amino acids (Sathisaran and Balasubramanian 2020). The polypeptide structure enables it to bond with oppositely charged groups (Jaison et al. 2020; Muhoza et al. 2019). Gelatin has been used alone or together with other biopolymers to be compatible with bioactive compounds (Amani et al. 2022; Muhoza et al. 2019; Sahoo et al. 2015). Compared to gelatin alone, gelatin‐based nanocomposites have superior physical and chemical properties such as tensile strength, conductivity, antimicrobial properties, water permeability properties, and inhibitory properties (Yang et al. 2021). Gelatin has been used alone or together with other biopolymers in encapsulating various compounds such as thyme essential oil (Vafania et al. 2019), the polyphenolic antioxidants of *Momordica charantia* fruit (Torkamani et al. 2018), pine honey (Parin et al. 2021), and curcumin (Wang et al. 2020).

Protein carriers are prone to degradation in gastric fluid, but the use of protein–polysaccharide carriers can provide a solution to protect sensitive bioactive compounds, reduce the rate of degradation of biopolymers, control the release rate of core materials, and achieve more successful applications (Dajic Stevanovic et al. 2020).

In the food industry, there has recently been increased interest among researchers in using new types of polysaccharides, such as gums, to encapsulate different compounds. Gums are hydrocolloid biopolymers that have various functional groups in their structure (Jaison et al. 2020). Many gums were used for availability encapsulation, such as Azivash (Hoseyni et al. 2021), Arabic (Eghbalifam et al. 2020; Serio et al. 2021; Silvestri et al. 2019), xanthan (Shekarforoush et al. 2018), tragacanth (Ranjbar‐Mohammadi et al. 2016; Ranjbar‐Mohammadi and Bahrami 2016), and guar (Aman Mohammadi et al. 2019) gum.

Sweet almond gum is one of these ingredients. Almond gum is a polysaccharide with a high molecular weight that exhibits excellent solubility and is well‐suited for the process of electrospinning due to its cheapness, availability, high water absorption, nontoxic and biodegradable nature, and stability in acidic conditions (Muhammad et al. 2019; Rezaei et al. 2016). Electrospun nanofibers derived from almond gum present a favorable option for the preservation and enhancement of the stability of delicate compounds (Rezaei and Nasirpour 2019).

Gelatin and almond gum are biopolymers that are widely available and inexpensive, and possess encapsulation properties. These polymers can be used in combination for the production of a system that allows for the controlled release of bio components in response to digestive conditions.

Therefore, this paper utilized sweet almond gum, an anionic polymer, to form complex compounds with gelatin for the encapsulation of olive leaf polyphenols. Based on the topics discussed above, the main contributions of this paper are as follows:

1. Simultaneous use of sweet almond gum and gelatin as wall material in electrospinning.

2. Encapsulating olive leaf polyphenols in electrospun nanofibers of sweet almond gum/gelatin.

## 2 Materials and Methods

### 2.1 Materials

Gelatin powder (250 g) manufactured by South Korea Samchan Company was purchased. The sweet almond gum used in this study was obtained from secretions collected from the trunks and branches of *Amygdalus communis* L. trees in Kermanshah province, Iran. The leaves of the olive tree ( *Olea europaea* L.) belonging to the Oleaceae family were obtained from Khorramabad, Iran, and its polyphenols were obtained in the laboratory and kept in a refrigerator (4°C) until use. Glacial acetic acid (C_2_H_4_O_2_) with a purity of 96% made by Merck, distilled water, acetone (O_6_H_3_C), n‐hexane (C_6_H_14_) from Merck, chloroform (C_1_H_1_C_l3_), and ethyl acetate (C_4_H_8_O_2_) were purchased.

### 2.2 Preparation and Extraction of Polyphenol

The leaves of the olive tree ( *Olea europaea* L.) belonging to the Oleaceae family (Özcan and Matthäus 2016) were obtained from Khorramabad city and after washing, its polyphenols were extracted with the method provided by Gariboldi et al. (1986). In this way, first, 400 g of ground olive leaves was divided into four parts, and then 500 mL of 96% ethanol was added to each. After closing the lid of the container to prevent light contact, it was wrapped around the aluminum foil container and kept for 3 days and nights. The container containing olive leaves and ethanol was placed on the heater stirrer. Then, the contents of the containers were filtered using filter paper, and the resulting extract was transferred to a rotary evaporator (Heidolph Laborota 4001, Korea) to separate ethanol at 40°C. To continue the extraction, a water–acetone solution was added to the remaining material at a ratio of 1:1 (50 mL of water and 50 mL of acetone). Then, it was washed four times with hexane. In this way, in the first order, 50 mL of hexane was added to the sample, and the contents were transferred to the decanter for two phases. After a few minutes, two phases were formed in the decanter, where the hexane and impurities were discarded, and the second phase entered the second stage of washing. In the second stage, 50 mL of hexane was added, and washing continued for four stages (in each stage, the decanter was washed to reduce the impurities). In continuation of the extraction, chloroform solvent was used. In the first step of washing, 50 mL of chloroform was used, and washing with chloroform was done up to four times. It is necessary to explain that chloroform should be discarded, despite hexane. Washing continued four times with ethyl acetate. After the fourth step of washing with ethyl acetate, what remained contained polyphenols, which were again transferred to the rotary under vacuum to separate the solvents. The obtained extract was stored in a refrigerator (4°C) until use.

### 2.3 Electrospinning Process

First, gelatin solutions (25% w/v) in different device conditions (applied voltage in the range of 20–30 kV, feeding speed of the electrospinning solution in the range of 0.25–0.75 mL/h, the distance between the needles, and the head and surface area of nanofiber collection in the range of 10–20 cm) were prepared to obtain optimal nanofibers from gelatin using Design Expert Software version 13 (Table 1), and then SEM images were taken.

**TABLE 1:** Gelatin pretests for the preparation of optimal nanofibers.

Acetic acid (mL)/Distilled water (mL)/gelatin (g)	Needle diameter (mm)	Foil size (cm^2^)	Voltage (KV)	Diameter (mm)	Rate (mL/h)	Max volume (mL)	Start pos (mm)	End pos (mm)	Speed (mm/min)	Distance (cm)	Scan Pos (mm)	Drum (rpm)
7.5/2.5/2.5	16	5*5	25	13.0	0.50	3.0	250	250	0	14	250	Off
7.5/2.5/2.5	16	5*5	20	13.0	0.50	3.0	250	250	0	14	250	Off
7.5/2.5/2.5	16	5*5	17	13.0	0.50	3.0	250	250	0	14	250	Off
7.5/2.5/2.5	16	24*15	25	13.0	0.50	3.0	250	250	0	14	250	Off
7.5/2.5/2.5	16	22*20	25	13.0	0.50	3.0	250	250	0	14	250	Off
7.5/2.5/2.5	16	5*5	25	13.0	0.50	3.0	250	250	0	15	250	Off
7.5/2.5/2.5	16	15*20	25	13.0	0.50	3.0	250	250	0	15	250	Off
7.5/2.5/2.5	16	5*5	25	13.0	1.00	3.0	250	250	0	15	250	Off
7.5/2.5/2.5	16	15*20	25	13.0	1.00	3.0	250	250	0	15	250	Off
7.5/2.5/2.5	16	15*20	25	13.0	0.50	3.0	250	250	0	20	250	Off

Given that nanofibers were not produced from almond gum solutions alone, a mixed solution of almond gum/gelatin in different ratios was investigated and electrospinning was performed. Initially, almond gum solutions (10% w/w, 11% w/w, 12% w/w, 13% w/w, and 14% w/w) and gelatin at a constant concentration (25% w/w) with different ratios (50:50, 25:75, 30:70, and 20:80) without adding polyphenols were electrospun under ambient conditions (temperature 25°C and relative humidity 30%) and different machine conditions (voltage, distance, and feeding speed). The gum solution with a concentration of 10% w/w and the gelatin solution with a constant concentration of 25% w/w with a ratio of 50:50 were selected as the desired samples. Then, the desired sample was used as a coating for microencapsulation of olive leaf polyphenol extract at concentrations of 0%, 5%, 10%, and 20% (Table 2).

**TABLE 2:** Pretests of gelatin/almond gum solution (in different concentrations) to prepare suitable nanofibers.

Acetic acid (mL)/distilled water (mL)/gelatin (g)	Acetic acid (mL)/distilled water (mL)/Sweet almond gum (g)	Needle diameter (mm)	Foil size (cm^2^)	Voltage (KV)	Diameter (mm)	Rate (mL/h)	Max volume (mL)	Start pos (mm)	End pos (mm)	Speed (mm/min)	Distance (cm)	Scan Pos (mm)	Drum (rpm)
7.5/2.5/2.5 50%	7.5/2.5/1.0 50%	16	15*20	25	13.0	0.50	3.0	250	250	0	15	250	Off
7.5/2.5/2.5^a^ 50%	7.5/2.5/1.0 50%	16	5*5	25	13.0	0.50	3.0	250	250	0	15	250	Off
7.5/2.5/2.5 75%	7.5/2.5/1.0 25%	16	5*5	25	13.0	0.50	3.0	250	250	0	15	250	Off
7.5/2.5/2.5 50%	7.5/2.5/1.1 50%	16	5*5	25	13.0	0.50	3.0	250	250	0	15	250	Off
7.5/2.5/2.5 70%	7.5/2.5/1.1 30%	16	5*5	25	13.0	0.50	3.0	250	250	0	15	250	Off
7.5/2.5/2.5 50%	7.5/2.5/1.2 50%	16	5*5	25	13.0	0.50	3.0	250	250	0	15	250	Off
7.5/2.5/2.5 80%	7.5/2.5/1.2 20%	16	5*5	25	13.0	0.50	3.0	250	250	0	15	250	Off
7.5/2.5/2.5 50%	7.5/2.5/1.3 50%	16	5*5	25	13.0	0.50	3.0	250	250	0	15	250	Off
7.5/2.5/2.5 70%	7.5/2.5/1.3 30%	16	5*5	25	13.0	0.50	3.0	250	250	0	15	250	Off
7.5/2.5/2.5 50%	7.5/2.5/1.4 50%	16	5*5	25	13.0	0.50	3.0	250	250	0	15	250	Off
7.5/2.5/2.5 70%	7.5/2.5/1.4 30%	16	5*5	25	13.0	0.50	3.0	250	250	0	15	250	Off

^a^ Optimal sample.

According to the SEM images (at three magnifications of 1000, 2000, and 5000), a sample made of 10% gum and 25% gelatin (50:50 ratio) with optimal voltage (25 kV), distance (150 mm), and feeding speed (0.5 mL/h) is the optimal sample (without knots and uniform).

Finally, olive leaf polyphenol with different concentrations (0%, 5%, 10%, and 20% by volume) was mixed with sweet almond gum/gelatin solution (50:50) and injected into a single‐axis electrospinning machine (ES1000 model, FNM Co., Iran). Then, SEM and viscosity images were taken from the obtained nanofibers. The electrospinning process was carried out for optimal gelatin/almond gum solutions (50:50) at an optimal voltage (25 kV), distance (150 mm), and feeding rate (0.5 mL/h). The electrospinning process was performed for 60 min for each sample (Miri et al. 2016). All solutions (polyphenol, almond gum, and gelatin) were prepared with acetic acid (70% v/v).

### 2.4 Atomic Force Microscope

The surface morphology of the fibers was examined using an AFM device (Ara Research Company, Tehran, Iran). The images were obtained in a noncontact mode in the air using commercial silicon consoles (Ara Research Company) with a resonance frequency of 180 kHz (Hosseini et al. 2021).

### 2.5 X‐Ray Diffraction

X‐ray diffraction analysis was conducted to evaluate the physical state of polyphenols in gelatin/almond gum electrospun fibers. The samples were analyzed using an X‐ray diffractometer within the range of 2θ = 4 to 40°, with a scanning speed of 0.05°/min, and Cu Kα radiation (1.54 Å) at a voltage of 40 kV and a current of 30 mA (Khammari et al. 2025). The X‐ray diffraction pattern was recorded at a wavelength of 0.154 nm.

### 2.6 Fourier Transform Infrared Spectroscopy

FTIR was employed to investigate the interaction between olive leaf polyphenols and gelatin/almond gum. The FTIR spectrum was recorded using a spectrophotometer (Thermo Nicolet AVATAR, USA) within the wavenumber range of 500–4000 cm^−1^ with a resolution of 4 cm^−1^ and a scanning distance of 12 cm (Bumedi et al. 2023). An average of 64 scans per sample was recorded.

### 2.7 Thermometric Analysis

The degradation behavior of fibers was studied using a TGA analyzer. Thermometric data were recorded under a nitrogen gas atmosphere with a heating rate of 10°C/min, within the range from room temperature to 800°C.

### 2.8 Encapsulation Efficiency

To measure the encapsulation efficiency of polyphenols in gelatin/almond gum electrospun fibers, 20 mg of nanofibers containing polyphenols was separated and immersed in 10 mL of acetic acid solvent separately for each concentration for a few minutes. Then, the absorbance of the samples was evaluated using a spectrophotometer at a wavelength of 254 nm (Gold Spectrumlab 54, USA). The actual amount of polyphenols was loaded and the encapsulation efficiency was calculated. The polyphenols in acetic acid were quantified by a calibration curve (*R* ^2^ = 0.9902) by having various amounts of polyphenols dissolved in acetic acid. Efficiency was calculated as follows:

(1) $$\text{encapsulation efficiency}=\frac{\left(A-B\right)}{A}*100$$

where *A* and *B* show the concentration of total polyphenol and free polyphenol in the solution, respectively (Hosseini et al. 2021).

### 2.9 Rheological Properties

The viscosity of the almond gum and gelatin solutions was measured separately; then, the viscosity of the mixture of almond gum/gelatin solution was measured, and finally, the viscosity of the almond gum/gelatin/polyphenol solutions at different concentrations (0%, 5%, 10%, and 20%) was measured at different shear speeds by rheometer with two parallel plates with a plate diameter of 25 mm and a distance of 1 mm was measured at a temperature of 25°C, which was done at cutting speeds (0.1–1000 s^−1^). Finally, to investigate the sample flow behavior of raw materials (gelatin, gum, and polyphenol in concentrations of 5%, 10%, and 20%) and electrospun solutions, two Newtonian and power law models were used, whose equations are as follows:

(2) $$\tau =\mu \dot{\gamma }$$

(3) $$\tau =k{\dot{\gamma }}^{n}$$

where *τ* is the shear stress (Pa), shear rate (1^−s^), *μ* is the viscosity of Newtonian fluids (Pa.s), *k* is the consistency coefficient (Pa.s^n^), and *n* is the power law index (dimensionless).

### 2.10 Polyphenol Release

To check the amount and release method of polyphenol compounds, the method of Charpashlo et al. (2019) was used with some changes. Thus, phosphate buffers with pH = 6.8, pH = 2.5, and pH = 7 were prepared as simulated salivary fluid (SSF), simulated gastric fluid (SGF), and simulated intestinal fluid (SIF), respectively. In the oral phase, the first 0.02 g of fibers containing polyphenols was combined with 20 mL of SSF, then placed in a shaker incubator for 10 min under temperature conditions of 37°C and shear speed of 100 rpm to simulate oral conditions. To measure the amount of release in a time interval of 2 min, a sample of 1 mL was taken. In the gastric phase, 20 mL of the mixture obtained from the oral phase along with fibers was combined with 20 mL of SGF, and the pH was adjusted to 2.5. The mixture was placed in a shaker incubator for 2 h under temperature conditions of 37°C and a shear speed of 100 rpm. Also, to determine the amount of release in the gastric phase, 1‐mL sampling was done at different time intervals of 2, 5, 10, 20, 40, 80, and 120 min. In the intestinal phase, 30 mL of the mixture obtained from the gastric phase along with fibers was transferred to a 100‐mL glass beaker and placed in a shaker incubator. The pH of the mixture was adjusted to 7, and 1.5 mL of SIF was added to it. It was kept for 2 h in the incubator shaker under temperature conditions of 37°C and a shear speed of 100 rpm (Minekus et al. 2014; Zhang et al. 2016). Also, sampling was done to determine the amount of release in the intestine as well as the gastric phase. Considering that the wavelength of olive leaf polyphenol is 254 nm, the absorption of the samples was measured at this wavelength using a spectrophotometer. The test was repeated twice. The amount of released polyphenol was calculated using the standard curve at all three pH levels.

## 3 Results and Discussion

### 3.1 Morphology and Diameter of Electrospun Fibers

**FIGURE 1:**
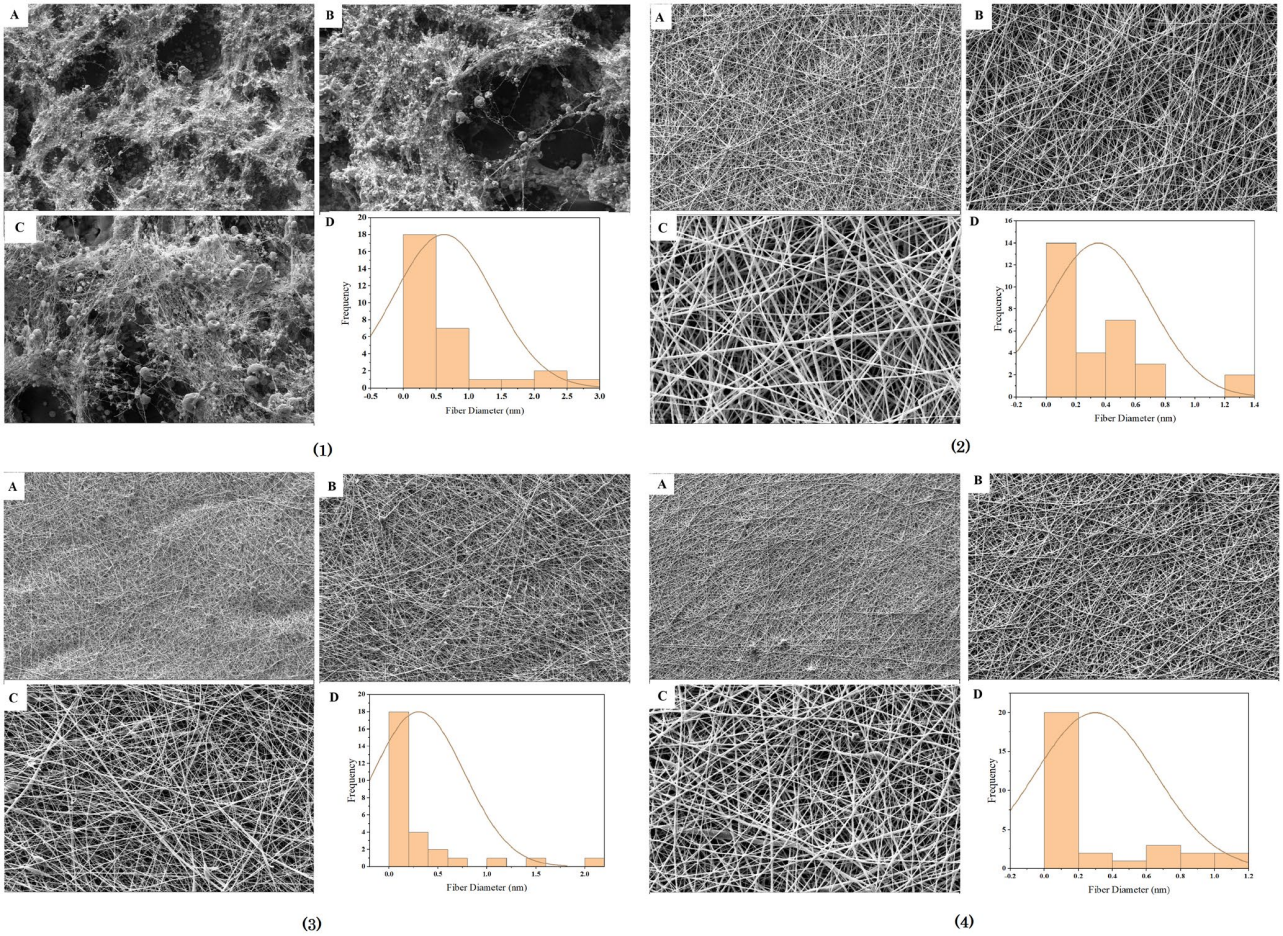
Scanning electron microscope images of fibers obtained from polyphenol‐free almond gum/gelatin electrospinning (1), with 5% (2), 10% (3), and 20% (4) polyphenol with magnification, × 1000 (A), × 5000 (C), × 2000 (B), histogram (D).

Figure 1 shows the morphology of electrospun fibers with different polyphenol concentrations (0%, 5%, 10%, and 20%).

As it was known in Figure 1 (1), almond gum/gelatin nanofibers without polyphenols have knots and are not uniform, but almond gum/gelatin nanofibers with polyphenols, 5% (Figure 1 (2)), 10% (Figure 1 (3)), and 20% (Figure 1 (4)) were knotless and uniform. This provides evidence that the polyphenols have been efficiently coated.

The results of the average diameter of nanofibers (Table 3) showed that with the increase in concentration of olive leaf polyphenols, the average diameter of the fibers has increased. Increasing the concentration of olive leaf polyphenols in nanofibers led to an increase in viscosity. As a result, the number of molecular entanglements in the solution increased, which contributed to the increase in the diameter of the electrospun fibers (Heydari‐Majd et al. 2019; Miri et al. 2021; Rezaeinia et al. 2020; Teo and Ramakrishna 2006). The electrical conductivity of solutions can be another factor that affects the behavior of fibers (Rezaeinia et al. 2020). As shown in Table 3, the electrical conductivity of solutions decreased with increasing polyphenol concentration (Tavassoli‐Kafrani et al. 2018; Teilaghi et al. 2020). Lowering the electrical conductivity reduced the charge density of the jet, resulting in decreased elasticity of the solution. This reduction in charge density caused the formation of fibers with larger diameters (Nayak et al. 2013; Ramakrishna et al. 2005; Rezaei et al. 2018).

**TABLE 3:** Average fiber diameter and electrical conductivity of sweet almond gum/gelatin/polyphenol solutions.

Solutions	Average fiber diameter (nm)	Electrical conductivity (μS/cm)
Sweet almond gum/gelatin	111	485
Sweet almond gum/gelatin with 5% polyphenol	296	463.5
Sweet almond gum/gelatin with 10% polyphenol	340	442
Sweet almond gum/gelatin with 20% polyphenol	410	399

**FIGURE 2:**
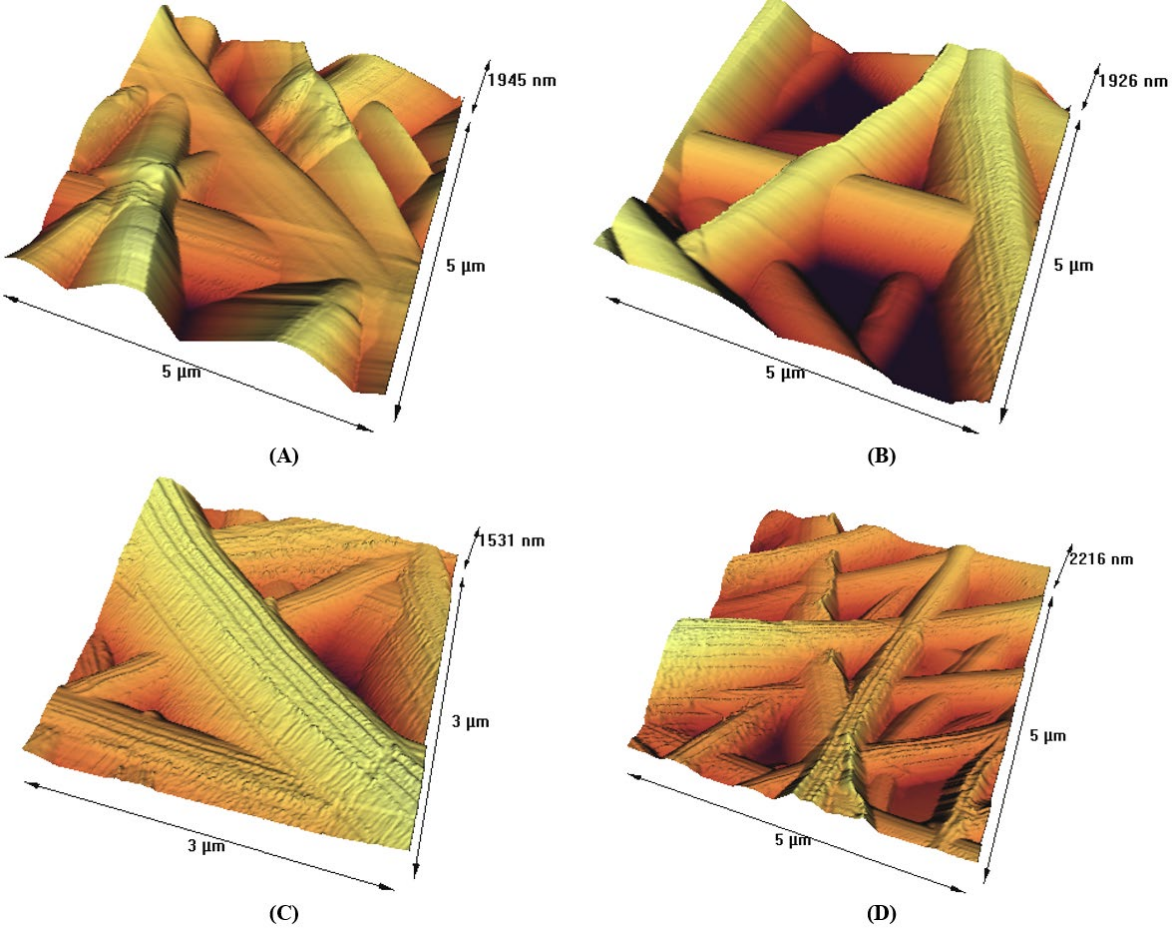
Atomic force microscope images of fibers obtained from electrospinning almond gum/gelatin nanofibers without polyphenol (A), with 5% (B), 10% (C), and 20% polyphenol (D).

Polymer solutions used in electrospinning can produce fibers with various shapes, including beaded and nonbeaded fibers, as well as tubular, flat ribbons, ribbons with other shapes, and fibers that were separated longitudinally from larger fibers (Koombhongse et al. 2001; Ramakrishna et al. 2005). AFM is a technique used to characterize the surface topography of electrospun fibers. AFM images of gelatin/gum electrospun fibers with polyphenols and without polyphenols are shown in Figure 2. It can be seen that the morphology of electrospun fibers was a completely uniform tube without any disorder. This finding is consistent with the research conducted by Torkamani et al. (2018). AFM images provided a better view of the tubular shape of the electrospun fibers compared to SEM images. It is suggested that the formation of tubular fibers is more likely to occur when a highly volatile solvent system, such as acetic acid, is used for electrospinning (Miri et al. 2021).

### 3.2 X‐Ray Diffraction

**FIGURE 3:**
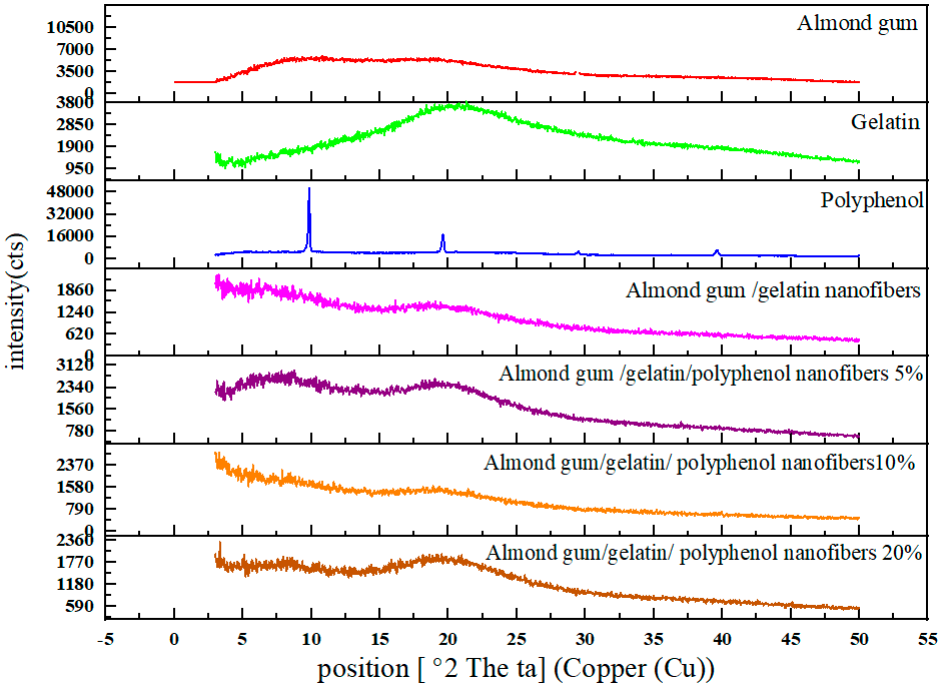
X‐ray diffraction patterns of almond gum, gelatin, polyphenol, almond gum/gelatin nanofibers, almond gum/gelatin/polyphenol nanofibers (5%, 10%, and 20%).

X‐ray diffraction test examines the physical state of polyphenols in electrospun fibers and the crystal structure of the samples. Figure 3 shows the X‐ray diffraction patterns of gelatin powder, almond gum powder, olive leaf polyphenol, and almond gum/gelatin with different polyphenol concentrations (0%, 5%, 10%, and 20%). Almond gum showed a broad band in the approximate region of 20° and 9°, and a narrow indicator band was seen in the approximate region of 29°. The X‐ray diffraction pattern of almond gum showed a completely amorphous structure. The X‐ray diffraction pattern of gelatin showed a broad peak at 20°, which indicated the distance between the polypeptide branches in the gelatin chain (Panzavolta et al. 2010). Moreover, narrow index bands were seen at 10°, 19.5°, 29.5°, and 39.5°. Therefore, a completely amorphous structure was shown for gelatin. As can be seen, almond gum/gelatin nanofibers had a completely amorphous structure. However, sharp peaks were seen in polyphenol, which showed that polyphenol has a crystalline structure. In almond gum/gelatin nanofibers encapsulated with different concentrations of polyphenol (5%, 10%, and 20%), no sharp peaks of polyphenol were seen, which indicated the good mixing of polyphenol with almond gum and gelatin. In other words, polyphenol was well dissolved in the structure of nanofibers of almond gum and gelatin, and it was no longer crystalline and has become amorphous.

### 3.3 Thermogravimetric Analysis

A thermogravimetric analyzer was utilized to investigate the degradation behavior and thermal decomposition of almond gum/gelatin/polyphenol nanofibers.

**FIGURE 4:**
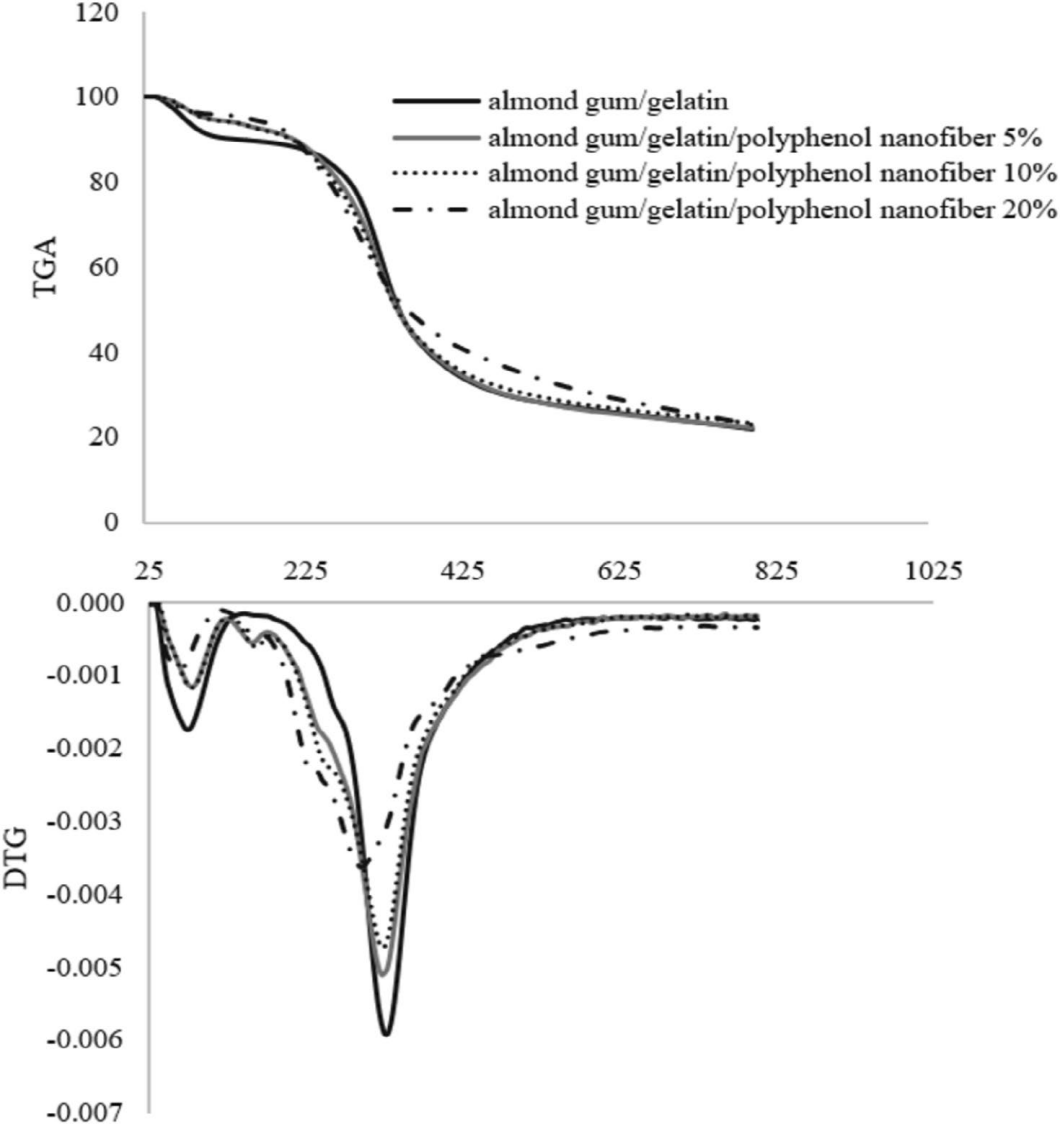
Graphs of thermal analysis of fibers obtained from electrospinning of almond gum/gelatin with different concentrations (0%, 5%, 10%, and 20%) of polyphenol.

Figure 4 shows the TGA curves of almond gum/gelatin/polyphenol nanofibers (0%, 5%, 10%, and 20%). In the TGA curve of almond gum/gelatin without polyphenol, weight loss occurs in two distinct stages. The first stage of the weight loss (10%), attributed to moisture evaporation, extends up to 140.27°C. The second stage of the weight loss (68.15%), marked by a sharp slope, occurred from 140.27°C to 800.45°C and corresponds to the degradation of gelatin and almond gum. Rezaei et al. (2016) also showed that the main degradation of almond gum starts at 260°C. Moreover, Gautam et al. (2013) illustrated that the degradation of gelatin was recorded in two stages. In the first stage, the weight loss from 50°C to 150°C can be described as due to moisture evaporation, while the second stage, which starts at about 250°C and is completed at about 600°C, is the main zone of thermal degradation and is related to a complex process involving protein chain breakage and peptide bond rupture.

The TGA curve of almond gum nanofibers/gelatin/polyphenol (5%) exhibited three distinct stages of weight loss. The first stage, with a weight loss of 5.63%, occurred up to 118.13°C. This was followed by a second stage, with a weight loss of 2.45%, extending up to 181.10°C. The final stage, accounting for a weight loss of 68.50%, continued from 181.10°C to 800.39°C. Similarly, the TGA curve of almond gum/gelatin/polyphenol nanofibers (10%) displayed an initial weight loss of 5.35% up to 118.61°C, succeeded by a second weight loss of 2.33% up to 181.09°C. The main stage of weight loss, representing 69.99%, occurred from 181.09°C to 800.41°C. The TGA curve of almond gum/gelatin/polyphenol nanofibers (20%) exhibited an initial weight loss of 4.20% up to 118.17°C. This was followed by the main weight loss stage, which represented 72.90% and spanned from 118.17°C to 800.20°C.

As shown in Figure 4, the weight loss observed at temperatures below 118°C in samples containing polyphenol and below 140°C in samples without polyphenol is attributed to the evaporation of moisture.

Erdogan et al. (2015) also showed that the existence of olive leaf extract demonstrated a distinct decomposition pattern within a comparable temperature range. The initial mass loss was attributed to the evaporation of water, followed by a further reduction of 55% at 180°C. Beyond 300°C, the rate of degradation decreased significantly. Polyphenols, constituting 22% of the mass, remained stable after analysis at 600°C, indicating their thermal stability. It was estimated that the complete degradation of olive leaf extract occurs at temperatures above 800°C. The interaction of the aqueous acetic acid phase of olive leaf polyphenols, gelatin, and sweet almond gum potentially caused irreversible structural modifications, leading to improved mechanical properties and increased resistance to thermal degradation compared to pure materials. Wall material could generate numerous robust hydrogen bonds and enhance hydrogen bond formation via electrostatic fields, leading to a more stable system and an elevation in its degradation temperature. Erdogan et al. (2015) and Jiang et al. (2025) showed better thermal stability and mechanical properties in the presence of polyphenols in the electrospun zein fibers and the double‐layer composite fiber film, respectively.

Similar results by Amani et al. (2022), Jannasari et al. (2019), and Gharanjig et al. (2020) on rosemary essential oil, vitamin D, and natural anthocyanins encapsulation, respectively, have also shown that encapsulating sensitive compounds with complex biopolymers is a valuable approach to enhance their thermal stability.

### 3.4 Interaction Between Polyphenol and Gelatin and Sweet Almond Gum

**FIGURE 5:**
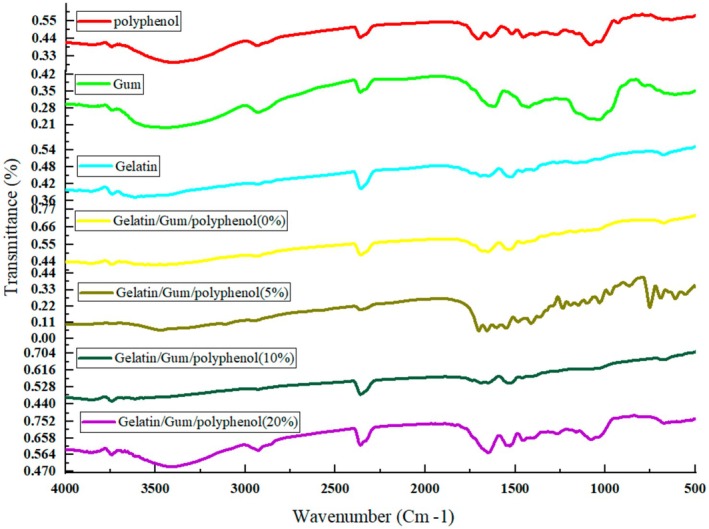
Infrared spectroscopic analysis of fibers obtained by electrospinning olive leaf polyphenol, sweet almond gum powder, gelatin powder, gelatin nanofibers/almond gum, almond gum nanofibers/gelatin/polyphenol (5%, 10%, and 20%).

Possible interactions between gelatin and almond gum and polyphenol in electrospun nanofibers were investigated by FTIR spectrum. Analyses were performed in the range of 500–4000 cm^−1^ using FTIR spectrometer. The FTIR spectrum of gelatin (Figure 5) showed a broad peak at about 3305 cm^−1^ related to the O—H stretching vibration (Liu et al. 2021). The characteristic absorption bands of the gelatin scaffold around 1650 cm^−1^, 3291–3298 cm^−1^ (amide I) (Derkach et al. 2020; Zhang and Zhang 2018), 1540 cm^−1^ (amide II) (Ahlawat et al. 2019), and 1242 cm^−1^ (amide III) (Wang et al. 2020) which were related to C—O bond stretching, N—H bond bending, and C—H bond stretching. Moreover, gelatin showed peaks at 3100–3500 cm^−1^, which were related to N—H stretching vibrations and hydrogen bonding.

The FTIR spectrum of the almond gum powder sample (Figure 5) showed a broad band in the range of 3330, 1602, and 2924, 2862 cm,^−1^ which was related to the stretching vibration of hydroxyl, the asymmetric stretching of carboxyl, and the symmetric and asymmetric C—H_2_ groups in almond gum. The bands shown in the range of 500–1500 cm^−1^ correspond to the specific region of carbohydrates (Rezaei et al. 2016).

The characteristic bands of polyphenols in Figure 5 were 3398.19, 2930.08 (amide I), 1516 cm^−1^ (amide II), and 1455.77 cm,^−1^ which were related to intermolecular hydrogen bonds, methylene groups (C—H) in phenolic rings, C—O bond stretching, N—H bond bending, and C—C bending in phenolic compounds, respectively (Soleimanifar et al. 2020; Torkamani et al. 2018).

Spectroscopic results of almond gum/gelatin nanofibers showed an interaction between the carboxyl group of almond gum and amino group of the gelatin because the bands of 2369.45, 1521.25, and 3448.99 cm^−1^ in almond gum have shifted to 2357.66, 1537.91, and 3505.01 cm^−1^, respectively. Similar results were reported by Amani et al. (2022). Moreover, the bands of 673.65, 1648.02, and 3738.24 cm^−1^ in gelatin were shifted to 674.32, 1651.34, and 3740.92 cm^−1^ in almond gum/gelatin nanofibers, which were due to the interaction between gelatin and almond gum. The intensity of bands 1537.91 and 1651.37 cm^−1^ in almond gum/gelatin nanofibers was more than almond gum powder, which was due to the overlap of gelatin bonds with almond gum.

Spectroscopic results of almond gum/gelatin/polyphenol nanofibers showed that the characteristic bands of 1455.77 and 2930.08 cm^−1^ were related to the aromatic rings of phenolic compounds (C—C bending) and methylene groups (C—H) in phenolic rings, which confirms the presence of polyphenols in the nanofibers.

The band of 1651.34 cm^−1^ in almond gum/gelatin nanofibers was, respectively, shifted to 1691.32 cm^−1^, 1656.93 cm^−1^, and 1651.37 cm^−1^ in 5%, 10%, and 20% in almond gum/gelatin/polyphenol nanofibers. Moreover, the characteristic band of 3398.19 cm^−1^ in polyphenols that related to intermolecular hydrogen bonds shifted to 3469.11, 3451.12, and 3411.61 cm^−1^ in 5%, 10%, and 20% polyphenol nanofibers. These were due to interactions between the polyphenols and the encapsulating material, which confirms the successful encapsulation of olive leaf polyphenols.

The FTIR spectra of the final nanofibers showed significant differences in the regions 800–1200 cm^−1^. The corresponding peak intensity increased with the addition of polyphenol concentration, which is likely due to vibrational C—C, C—O stretching vibration, and bending vibration of the benzene group in polyphenols. This observation demonstrated a concentration‐dependent relationship between polyphenol content and the strength of molecular interactions within the nanofiber matrix.

### 3.5 Encapsulation Efficiency

The encapsulation efficiency shows the effectiveness of the capsule in preserving the encapsulated compounds, and the extent of this phenomenon relies significantly on the compatibility between the polymer matrix and the active compounds (De Dicastillo et al. 2019). Results showed that the encapsulation efficiency was increased significantly by increasing polyphenol concentration, so that the encapsulation efficiency of olive leaf polyphenol in almond gum/gelatin electrospun fibers was 87.83%, 90.51%, and 91.78%, respectively, for the levels of 5%, 10%, and 20% loading of polyphenols. Higher polyphenol concentrations can enhance interactions (such as hydrogen bonding, hydrophobic interactions, or electrostatic attractions) between the polyphenols and the encapsulating material. These interactions stabilize the encapsulated structure and improve efficiency (Mihaly Cozmuta et al. 2024; Pedrali et al. 2023). Moreover, at low concentrations, the polyphenols work as individual molecules that can conjugate exclusively with proteins such as gelatin, while at higher concentrations of polyphenols, they might interact with proteins as an aggregate as well as gradually coat the peptides to dimerize and create an insoluble complex and improve efficiency (Muntaha et al. 2025; Pianet et al. 2008; Poklar Ulrih 2017).

Soleimanifar et al. (2020) encapsulated olive leaf extract in whey protein fibers and reported an increase in efficiency with an increase in the polyphenol concentration. Manafi Dizajyekan et al. (2021) encapsulated phenolic compounds of olive leaf extract in the form of nanoliposomes and reported 74%–78% retention.

### 3.6 Rheology Test

**FIGURE 6:**
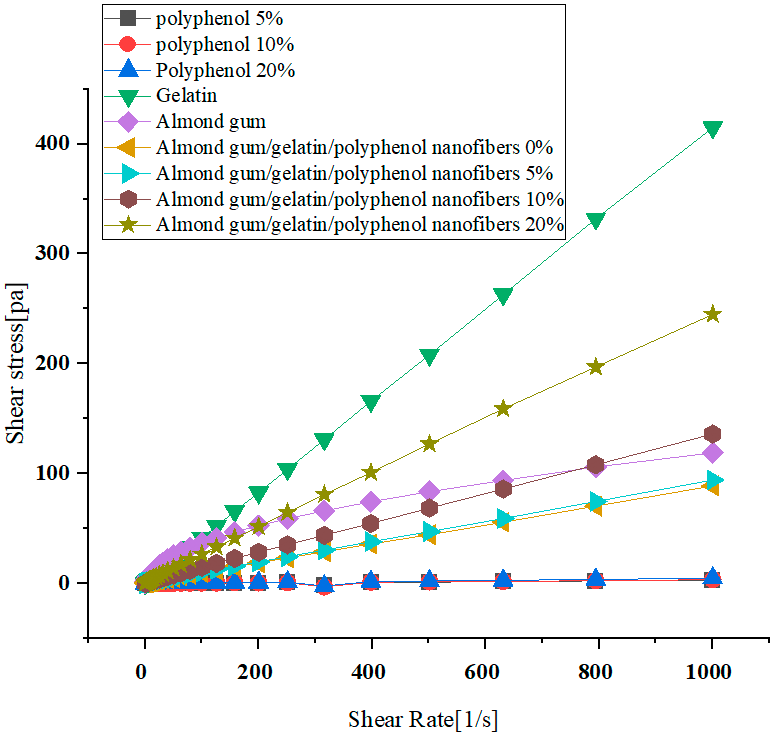
Graphs of shear stress versus shear rate of almond gum, gelatin, polyphenol (5%, 10%, and 20%), and almond gum/gelatin/polyphenol nanofibers (0%, 5%, 10%, and 20%).

Figure 6 shows the curves of shear stress versus shear rate. As shown, with the increase in shear rate, shear stress also increases in all samples.

In all the samples, except the almond gum solution (10% w/v), the most suitable predictive model for determining the rheological behavior was the Newtonian model (*R* ^2^ ≥ 0.99), which indicates its Newtonian behavior (Table 4). The linear relationship between shear stress and shear rate observed in Figure 6 also confirms the Newtonian behavior of the samples.

**TABLE 4:** Newtonian model parameters of samples with Newtonian fluid behavior at room temperature.

Solutions	μ (Pa.s)	*R* ^2^
Gelatin	0.4162	1
5% polyphenol	0.0028	0.99
10% polyphenol	0.0033	0.99
20% polyphenol	0.0047	0.99
Sweet almond gum/gelatin	0.0881	0.99
Sweet almond gum/gelatin with 5% polyphenol	0.0939	0.99
Sweet almond gum/gelatin with 10% polyphenol	0.1359	0.99
Sweet almond gum/gelatin with 20% polyphenol	0.2477	0.99

Table 4 shows the viscosity coefficient determined for Newtonian fluids. Increasing the polyphenol concentration has led to an increase in the viscosity of the nanofiber solutions. Unlike many hydrocolloid solutions, gelatin (25% w/v) showed Newtonian behavior. Atay et al. (2018) used the combination of gelatin and chitosan to encapsulate an active anthocyanin compound by electro‐spraying and reported the Newtonian behavior of pure gelatin solution. Also, Mohajeri et al. (2023) observed the Newtonian behavior of gelatin solution to investigate the production of gelatin nanofibers by the electrospinning method of Wulansari et al. (1998) related to the Newtonian behavior of gelatin solutions to the compact spatial arrangement of gelatin polymer chains.

According to Table 4, the high coefficient of determination related to the fit of the power law model with the rheological data of the gum solution showed that this solution has a non‐Newtonian behavior, which was confirmed by the nonlinear relationship between the shear stress and the shear rate (Figure 6). High molecular weight and entanglements and aggregations of polymer chains through hydrogen bonds were the reasons for the pseudoplastic behavior of almond gum (Vardhanabhuti and Ikeda 2006).

However, the presence of almond gum in the electrospun solutions was not able to change the flow behavior of the samples from Newtonian to non‐Newtonian (Table 5). The Power law index of almond gum solution was 0.67 (*n* < 1), which indicated its pseudoplastic behavior (shear thinning). When the Power law index approaches one, the fluid behavior becomes more similar to that of Newtonian fluids; whereas when this index approaches zero, the fluid behavior becomes more similar to that of non‐Newtonian fluids. As a result, the Newtonian behavior of electrospinning solutions may be attributed to the relatively high power law index of the gum solution and the Newtonian behavior of the 25% gelatin solution.

**TABLE 5:** Power law model parameters of sweet almond gum at room temperature.

Solution	*n*	*k* (Pa. s^n^)	*R* ^2^
Sweet almond gum	0.6778	1.5325	0.98

### 3.7 Polyphenol Release

**FIGURE 7:**
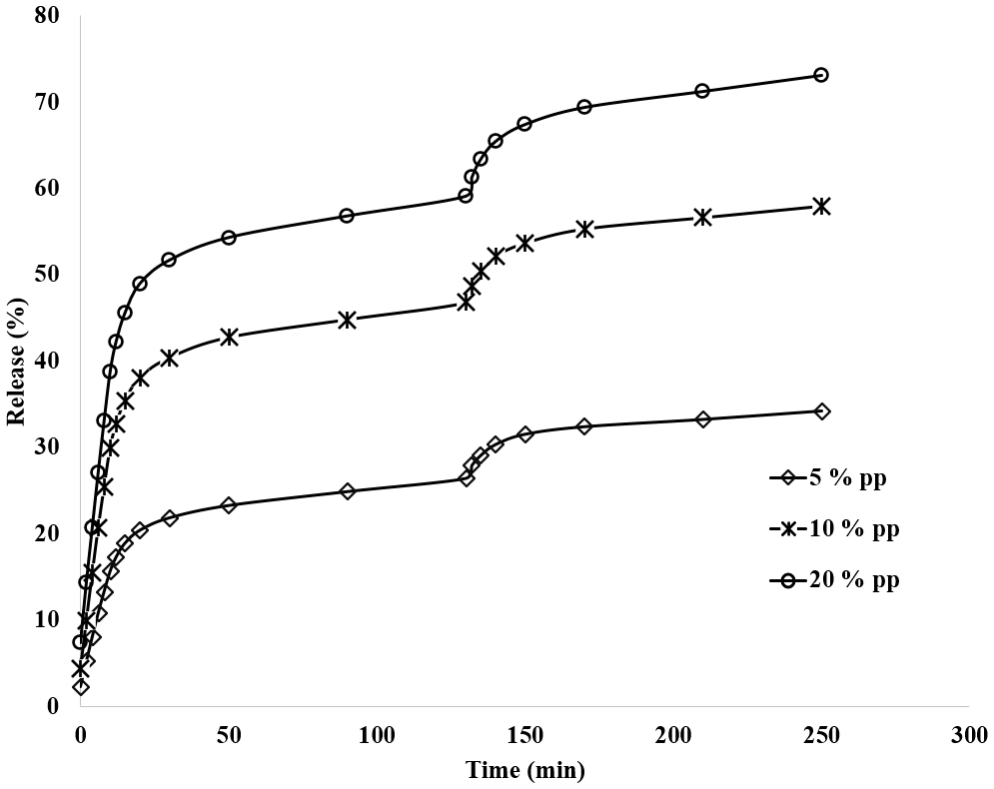
Olive leaf polyphenol release profile from gelatin/gum nanofibers in three concentrations used during the passage through the digestive tract.

The release rate of olive leaf polyphenols from gelatin/almond gum nanofibers at concentrations of 5%, 10%, and 20% in the digestive tract is shown in Figure 7. As shown, the release rate increased with the increase in time in all samples. Moreover, the release rate increased with the increase in polyphenol concentration at the same time; thus, the release rate was 34.20%, 57.94%, and 73.05% for concentrations of 5%, 10%, and 20%, respectively. It could be related to the increase in the kinetic speed of compounds with increasing concentration. Similar findings were reported by Agarwal and Murthy (2015), Khoshakhlagh et al. (2020), and Giese et al. (2018) on the releasing of mucoadhesive gastroretentive tablets, nanoencapsulated D‐limonene, and engineered nanomaterial, respectively.

In all the tested concentrations, the release rate is high in simulated saliva, which can be related to the absorption of water by the gum and gelatin at the beginning of the process and the increase in the flexibility of the nanofibers and the sufficient space for exit polyphenol provides nanofibers. A comparison of the release profiles of polyphenols in a simulated saliva and gastric condition revealed a significant reduction in polyphenol release upon exposure to the gastric conditions. This phenomenon can be attributed to the decrease in wall material solubility and the reduced permeability of compounds at lower pH levels. Moreover, the structure of the gum swells and the size of the particles increases with the increase of water absorption, and the exit path of polyphenol from the nanofibers becomes longer, and its release decreases (Siepmann and Göpferich 2001). Furthermore, as the compound was hydrated, a visible gel formed around the nanocapsules, which impeded the release of core molecules into the environment (Lamoudi et al. 2016). These findings align with those reported by Sanatkar et al. (2022) on daidzein‐loaded chitosan microcapsules.

Upon transitioning from the simulated gastric environment to the intestinal phase, the release rate decreases despite the increase in pH. This reduction is likely due to the decreased polyphenol content in the formulation, a lower concentration gradient, and ultimately, a reduced diffusion rate (Khoshakhlagh et al. 2018). Since the sample passed through the simulated salivary and gastric environments before entering the intestinal phase, the continuous pH changes across these three environments may also induce interactions within the nanofibers, leading to a lower release rate in the simulated intestinal environment and not releasing 100% of polyphenols during release processing.

Similar findings were reported by Lamoudi et al. (2016) and Khoshakhlagh et al. (2018) on sodium diclofenac from heterogeneous matrix tablets and nanoencapsulated D‐limonene with *Alyssum Homolocarpum* seed gum.

## 4 Conclusion

This study employed the electrospinning technique to encapsulate olive leaf polyphenols within sweet almond gum/gelatin composite nanofibers. SEM and AFM analyses revealed that the almond gum/gelatin/polyphenol nanofibers exhibited perfectly tubular morphology with a uniform structure, devoid of knots or irregularities. Notably, fiber diameters showed a concentration‐dependent increase with higher polyphenol content. FTIR spectroscopy confirmed both the molecular interaction between gelatin and almond gum components and the successful encapsulation of olive leaf polyphenol extract within the nanofiber matrix. XRD results showed that polyphenol with different concentrations in almond gum/gelatin nanofibers has an amorphous state. Additionally, the results of TGA showed that the first, second, and third stages of weight loss were related to the evaporation of moisture, degradation of polyphenols, and degradation of gelatin polymer and almond gum, respectively. The rheological analysis revealed that the nanofiber solutions exhibited Newtonian behavior, in contrast to the non‐Newtonian characteristics observed in the almond gum solution. Furthermore, the release rate demonstrated a concentration‐dependent enhancement, increasing from 34.20% to 73.05% with higher polyphenol concentrations. This study demonstrates that electrospun olive leaf polyphenol nanofibers present promising applications in both food and pharmaceutical industries by enhancing bioavailability, enabling controlled release, extending shelf life, and masking the undesirable taste of polyphenols.

## Author Contributions

**Shiva Geravand:** conceptualization (equal), data curation (equal), formal analysis (equal), writing – original draft (equal). **Sediqeh Soleimanifard:** conceptualization (lead), data curation (lead), formal analysis (lead), methodology (equal), project administration (lead), supervision (equal), writing – review and editing (lead). **Mohammad Amin Miri:** methodology (equal), supervision (equal). **Mahmoud Tavakoli:** methodology (equal). **Atefe Rezaei:** conceptualization (equal), methodology (equal).

## Conflicts of Interest

The authors declare no conflicts of interest.

## Data Availability

The data underlying this article will be shared on reasonable request to the corresponding author.

## References

[fsn370335-bib-0001] Agarwal, S. , and R. S. R. Murthy . 2015. “Effect of Different Polymer Concentration on Drug Release Rate and Physicochemical Properties of Mucoadhesive Gastroretentive Tablets.” Indian Journal of Pharmaceutical Sciences 77: 705–714. 10.4103/0250-474x.174993. 26997698 PMC4778230

[fsn370335-bib-0002] Ahlawat, J. , V. Kumar , and P. Gopinath . 2019. “ *Carica papaya* Loaded Poly (Vinyl Alcohol)‐Gelatin Nanofibrous Scaffold for Potential Application in Wound Dressing.” Materials Science & Engineering. C, Materials for Biological Applications 103: 109834. 10.1016/j.msec.2019.109834. 31349516

[fsn370335-bib-0003] Aman Mohammadi, M. , S. Ramazani , M. Rostami , M. Raeisi , M. Tabibiazar , and M. Ghorbani . 2019. “Fabrication of Food‐Grade Nanofibers of Whey Protein Isolate–Guar Gum Using the Electrospinning Method.” Food Hydrocolloids 90: 99–104. 10.1016/j.foodhyd.2018.12.010.

[fsn370335-bib-0004] Amani, F. , A. Rezaei , M. S. Damavandi , A. S. Doost , and S. M. Jafari . 2022. “Colloidal Carriers of Almond Gum/Gelatin Coacervates for Rosemary Essential Oil: Characterization and In‐Vitro Cytotoxicity.” Food Chemistry 377: 131998. 10.1016/j.foodchem.2021.131998. 34999451

[fsn370335-bib-0006] Atay, E. , M. J. Fabra , M. Martínez‐Sanz , L. G. Gomez‐Mascaraque , A. Altan , and A. Lopez‐Rubio . 2018. “Development and Characterization of Chitosan/Gelatin Electrosprayed Microparticles as Food Grade Delivery Vehicles for Anthocyanin Extracts.” Food Hydrocolloids 77: 699–710. 10.1016/j.foodhyd.2017.11.011.

[fsn370335-bib-0007] Basal, G. , G. D. Tetik , G. Kurkcu , O. Bayraktar , I. D. Gurhan , and A. Atabey . 2016. “Olive Leaf Extract Loaded Silk Fibroin/Hyaluronic Acid Nanofiber Webs for Wound Dressing Applications.” Digest Journal of Nanomaterials and Biostructures 11: 1113–1123.

[fsn370335-bib-0008] Bayraktar, O. 2018. “Silk Fibroin Nanofibers Loaded With Hydroxytyrosol From Hydrolysis of Oleuropein in Olive Leaf Extract.” Textile & Leather Review 1, no. 3‐4: 90–98. 10.31881/tlr.2018.vol1.iss3-4.p90-98.a9.

[fsn370335-bib-0009] Bumedi, F. , M. Aran , M. A. Miri , and E. Seyedabadi . 2023. “Preparation and Characterization of Zein Electrospun Fibers Loaded With Savory Essential Oil for Fruit Preservation.” Industrial Crops and Products 203: 117121. 10.1016/j.indcrop.2023.117121.

[fsn370335-bib-0010] Burgain, J. , C. Gaiani , M. Linder , and J. Scher . 2011. “Encapsulation of Probiotic Living Cells: From Laboratory Scale to Industrial Applications.” Journal of Food Engineering 104: 467–483. 10.1016/j.jfoodeng.2010.12.031.

[fsn370335-bib-0011] Charpashlo, E. , M. Mohebbi , and B. Ghorani . 2019. “Electro‐Encapsulation of Lycopene in Protein Microfiber Structure: Physicochemical and Bioaccessibility Characteristics.” Innovative Food Technologies 6: 467–481. 10.22104/jift.2018.2626.1623.

[fsn370335-bib-0012] Chen, M. J. , and K. N. Chen . 2007. “Applications of Probiotic Encapsulation in Dairy Products.” In Encapsulation and Controlled Release Technologies in Food Systems, vol. 83–112. Wiley. 10.1002/9780470277881.ch4.

[fsn370335-bib-0013] Dajic Stevanovic, Z. , E. Sieniawska , K. Glowniak , N. Obradovic , and I. Pajic‐Lijakovic . 2020. “Natural Macromolecules as Carriers for Essential Oils: From Extraction to Biomedical Application.” Frontiers in Bioengineering and Biotechnology 8: 563. 10.3389/fbioe.2020.00563. 32671026 PMC7330110

[fsn370335-bib-0014] De Dicastillo, C. L. , C. Piña , L. Garrido , C. Arancibia , and M. J. Galotto . 2019. “Enhancing Thermal Stability and Bioaccesibility of açaí Fruit Polyphenols Through Electrohydrodynamic Encapsulation Into Zein Electrosprayed Particles.” Antioxidants 8, no. 10: 464. 10.3390/antiox8100464. 31600875 PMC6826472

[fsn370335-bib-0015] de la Ossa, J. G. , S. Danti , J. E. Salsano , et al. 2022. “Electrospun Poly(3‐Hydroxybutyrate‐Co‐3‐Hydroxyvalerate)/olive Leaf Extract Fiber Mesh as Prospective Bio‐Based Scaffold for Wound Healing.” Molecules 27, no. 6208: 6208. 10.3390/molecules27196208. 36234738 PMC9570516

[fsn370335-bib-0016] de la Ossa, J. G. , A. Fusco , B. Azimi , et al. 2021. “Immunomodulatory Activity of Electrospun Polyhydroxyalkanoate Fiber Scaffolds Incorporating Olive Leaf Extract.” Applied Sciences (Switzerland) 11, no. 9: 4006. 10.3390/app11094006.

[fsn370335-bib-0017] Derkach, S. R. , N. G. Voronko , N. I. Sokolan , D. S. Kolotova , and Y. A. Kuchina . 2020. “Interactions Between Gelatin and Sodium Alginate: UV and FTIR Studies.” Journal of Dispersion Science and Technology 41: 690–698. 10.1080/01932691.2019.1611437.

[fsn370335-bib-0018] Eghbalifam, N. , S. A. Shojaosadati , S. Hashemi‐Najafabadi , and A. C. Khorasani . 2020. “Synthesis and Characterization of Antimicrobial Wound Dressing Material Based on Silver Nanoparticles Loaded Gum Arabic Nanofibers.” International Journal of Biological Macromolecules 155: 119–130. 10.1016/j.ijbiomac.2020.03.194. 32224167

[fsn370335-bib-0019] Erdogan, I. , M. Aksit , and O. Bayraktar . 2015. “Olive Leaf Extract as a Crosslinking Agent for the Preparation of Electrospun Zein Fibers.” Journal of Applied Polymer Science 132, no. 4: 41338. 10.1002/app.41338.

[fsn370335-bib-0020] Gariboldi, P. , G. Jommi , and L. Verotta . 1986. “Secoiridoids From *Olea europaea* .” Phytochemistry 25: 865–869. 10.1016/0031-9422(86)80018-8.

[fsn370335-bib-0021] Gautam, S. , A. K. Dinda , and N. C. Mishra . 2013. “Fabrication and Characterization of PCL/Gelatin Composite Nanofibrous Scaffold for Tissue Engineering Applications by Electrospinning Method.” Materials Science and Engineering C 33: 1228–1235. 10.1016/j.msec.2012.12.015. 23827565

[fsn370335-bib-0022] Gharanjig, H. , K. Gharanjig , G. Farzi , M. Hosseinnezhad , and S. M. Jafari . 2020. “Novel Complex Coacervates Based on Zedo Gum, Cress Seed Gum and Gelatin for Loading of Natural Anthocyanins.” International Journal of Biological Macromolecules 164: 3349–3360. 10.1016/j.ijbiomac.2020.08.218. 32882277

[fsn370335-bib-0023] Ghorani, B. , A. Alehosseini , and N. Tucker . 2016. “Electrospinning as a Novel Delivery Vehicle for Bioactive Compounds in Food Nanotechnology.” In Innovative Processing Technologies for Foods With Bioactive Compounds, 259–292. CRC Press. 10.1201/9781315371276-13.

[fsn370335-bib-0024] Giese, B. , F. Klaessig , B. Park , et al. 2018. “Risks, Release and Concentrations of Engineered Nanomaterial in the Environment.” Scientific Reports 8: 1–18. 10.1038/s41598-018-19275-4. 29371617 PMC5785520

[fsn370335-bib-0025] Heydari‐Majd, M. , H. Rezaeinia , M. R. Shadan , B. Ghorani , and N. Tucker . 2019. “Enrichment of Zein Nanofibre Assemblies for Therapeutic Delivery of Barije ( *Ferula gummosa* Boiss) Essential Oil.” Journal of Drug Delivery Science and Technology 54: 101290. 10.1016/j.jddst.2019.101290.

[fsn370335-bib-0026] Hoseyni, S. Z. , S. M. Jafari , H. Shahiri Tabarestani , M. Ghorbani , E. Assadpour , and M. Sabaghi . 2021. “Release of Catechin From Azivash Gum‐Polyvinyl Alcohol Electrospun Nanofibers in Simulated Food and Digestion Media.” Food Hydrocolloids 112: 106366. 10.1016/j.foodhyd.2020.106366.

[fsn370335-bib-0027] Hosseini, F. , M. A. Miri , M. Najafi , S. Soleimanifard , and M. Aran . 2021. “Encapsulation of Rosemary Essential Oil in Zein by Electrospinning Technique.” Journal of Food Science 86: 4070–4086. 10.1111/1750-3841.15876. 34392535

[fsn370335-bib-0028] Jaison, D. , G. Chandrasekaran , and M. Mothilal . 2020. “pH‐Sensitive Natural Almond Gum Hydrocolloid Based Magnetic Nanocomposites for Theragnostic Applications.” International Journal of Biological Macromolecules 154: 256–266. 10.1016/j.ijbiomac.2020.03.103. 32179113

[fsn370335-bib-0029] Jannasari, N. , M. Fathi , S. J. Moshtaghian , and A. Abbaspourrad . 2019. “Microencapsulation of Vitamin D Using Gelatin and Cress Seed Mucilage: Production, Characterization and In Vivo Study.” International Journal of Biological Macromolecules 129: 972–979. 10.1016/j.ijbiomac.2019.02.096. 30779987

[fsn370335-bib-0030] Jiang, J. , Z. Liu , Z. Wang , et al. 2025. “Physicochemical Properties of Protein‐Polysaccharide‐Polyphenol Bilayer Composite Film Prepared by Electrospinning Layer‐By‐Layer Assembly Technology.” Food Packaging and Shelf Life 47: 101441. 10.1016/j.fpsl.2025.101441.

[fsn370335-bib-0031] Khammari, S. , M. Aran , M. A. Miri , and H. Ahmar . 2025. “Development of Fennel Essential Oil‐Loaded Zein Electrospun Fibers for Antimicrobial and Postharvest Preservation of Tangerines.” Journal of Food Measurement and Characterization 19: 3535–3551. 10.1007/S11694-025-03200-1.

[fsn370335-bib-0032] Khoshakhlagh, K. , M. Mohebbi , and A. Koocheki . 2020. “Evaluation of Structural Properties and Release Behavior of Nanoencapsulated D‐Limonene With Alyssum Homolocarpum Seed Gum by Electrospraying.” Research and Innovation in Food Science and Technology 9: 11–26. 10.22101/jrifst.2019.09.17.e1013.

[fsn370335-bib-0033] Khoshakhlagh, K. , M. Mohebbi , A. Koocheki , and A. Allafchian . 2018. “Encapsulation of D‐Limonene in Alyssum Homolocarpum Seed Gum Nanocapsules by Emulsion Electrospraying: Morphology Characterization and Stability Assessment.” Bioactive Carbohydrates and Dietary Fibre 16: 43–52. 10.1016/j.bcdf.2018.03.001.

[fsn370335-bib-0034] Khoshnoudi‐Nia, S. , N. Sharif , and S. M. Jafari . 2020. “Loading of Phenolic Compounds Into Electrospun Nanofibers and Electrosprayed Nanoparticles.” Trends in Food Science and Technology 95: 59–74. 10.1016/j.tifs.2019.11.013.

[fsn370335-bib-0035] Koombhongse, S. , W. Liu , and D. H. Reneker . 2001. “Flat Polymer Ribbons and Other Shapes by Electrospinning.” Journal of Polymer Science, Part B: Polymer Physics 39: 2598–2606. 10.1002/polb.10015.

[fsn370335-bib-0036] Kriegel, C. , A. Arrechi , K. Kit , D. J. McClements , and J. Weiss . 2008. “Fabrication, Functionalization, and Application of Electrospun Biopolymer Nanofibers.” Critical Reviews in Food Science and Nutrition 48: 775–797. 10.1080/10408390802241325. 18756399

[fsn370335-bib-0037] Lamoudi, L. , J. C. Chaumeil , and K. Daoud . 2016. “Swelling, Erosion and Drug Release Characteristics of Sodium Diclofenac From Heterogeneous Matrix Tablets.” Journal of Drug Delivery Science and Technology 31: 93–100. 10.1016/j.jddst.2015.12.005.

[fsn370335-bib-0038] Liu, Y. , D. Wang , Z. Sun , F. Liu , L. Du , and D. Wang . 2021. “Preparation and Characterization of Gelatin/Chitosan/3‐Phenylacetic Acid Food‐Packaging Nanofiber Antibacterial Films by Electrospinning.” International Journal of Biological Macromolecules 169: 161–170. 10.1016/j.ijbiomac.2020.12.046. 33309663

[fsn370335-bib-0039] Manafi Dizajyekan, M. , M. H. Hadad Khodaparast , S. Azadmard‐Damirchi , H. Valizadeh , and F. Tabatabaei Yazdi . 2021. “Effects of Olive Leaf Extract Nanoliposomes on Physicochemical, Microbiological and Sensory Properties of Butter.” Food Processing and Preservation Journal 13: 45–55. 10.22069/ejfpp.2021.11156.1347.

[fsn370335-bib-0040] Mihaly Cozmuta, A. , A. Peter , C. Nicula , et al. 2024. “The Impact of Visible Light Component Bands on Polyphenols From Red Grape Seed Extract Powder Encapsulated in Alginate–Whey Protein Matrix.” Food Chemistry 23: 101758. 10.1016/j.fochx.2024.101758. PMC11639332 39679380

[fsn370335-bib-0041] Minekus, M. , M. Alminger , P. Alvito , et al. 2014. “A Standardised Static In Vitro Digestion Method Suitable for Food – An International Consensus.” Food & Function 5: 1113–1124. 10.1039/c3fo60702j. 24803111

[fsn370335-bib-0042] Miri, M. A. , M. B. Habibi Najafi , J. Movaffagh , and B. Ghorani . 2021. “Encapsulation of Ascorbyl Palmitate in Zein by Electrospinning Technique.” Journal of Polymers and the Environment 29: 1089–1098. 10.1007/s10924-020-01954-x.

[fsn370335-bib-0043] Miri, M. A. , J. Movaffagh , M. B. Habibi Najafi , M. N. Najafi , B. Ghorani , and A. Koocheki . 2016. “Optimization of Elecrospinning Process of Zein Using Central Composite Design.” Fibers and Polymers 17: 769–777. 10.1007/s12221-016-6064-0.

[fsn370335-bib-0044] Mohajeri, P. , A. Hematian Sourki , A. Mehregan Nikoo , and Y. N. Ertas . 2023. “Fabrication, Characterisation and Antimicrobial Activity of Electrospun *Plantago Psyllium* L. Seed Gum/Gelatine Nanofibres Incorporated With *Cuminum Cyminum* Essential Oil Nanoemulsion.” International Journal of Food Science & Technology 58, no. 4: 1832–1840. 10.1111/ijfs.16324.

[fsn370335-bib-0045] Muhammad, D. R. A. , V. Gupta , A. S. Doost , and K. Dewettinck . 2019. “Functionality of Xanthan and Almond Gum in Colloidal Shellac Nanoparticles Containing Cinnamon.” IOP Conference Series: Materials Science and Engineering 633: 012030. 10.1088/1757-899x/633/1/012030.

[fsn370335-bib-0046] Muhoza, B. , S. Xia , J. Cai , X. Zhang , E. Duhoranimana , and J. Su . 2019. “Gelatin and Pectin Complex Coacervates as Carriers for Cinnamaldehyde: Effect of Pectin Esterification Degree on Coacervate Formation, and Enhanced Thermal Stability.” Food Hydrocolloids 87: 712–722. 10.1016/j.foodhyd.2018.08.051.

[fsn370335-bib-0047] Muntaha, S. T. , A. Rakha , H. Rasheed , et al. 2025. “Polyphenol‐Protein Particles: A Nutraceutical Breakthrough in Nutrition and Food Science.” Journal of Agriculture and Food Research 19: 101641. 10.1016/j.jafr.2025.101641.

[fsn370335-bib-0048] Nayak, R. , R. Padhye , I. L. Kyratzis , Y. B. Truong , and L. Arnold . 2013. “Effect of Viscosity and Electrical Conductivity on the Morphology and Fiber Diameter in Melt Electrospinning of Polypropylene.” Textile Research Journal 83: 606–617. 10.1177/0040517512458347.

[fsn370335-bib-0049] Özcan, M. M. , and B. Matthäus . 2016. “A Review: Benefit and Bioactive Properties of Olive (*Olea Europaea* L.) Leaves.” European Food Research and Technology 243, no. 1: 89–99. 10.1007/s00217-016-2726-9.

[fsn370335-bib-0050] Panzavolta, S. , M. Gioffrè , M. L. Focarete , C. Gualandi , L. Foroni , and A. Bigi . 2010. “Electrospun Gelatin Nanofibers: Optimization of Genipin Cross‐Linking to Preserve Fiber Morphology After Exposure to Water.” Acta Biomaterialia 7: 1702–1709. 10.1016/j.actbio.2010.11.021. 21095244

[fsn370335-bib-0051] Parin, F. N. , P. Terzioğlu , Y. Sicak , K. Yildirim , and M. Öztürk . 2021. “Pine Honey–Loaded Electrospun Poly (Vinyl Alcohol)/gelatin Nanofibers With Antioxidant Properties.” Journal of the Textile Institute 112: 628–635. 10.1080/00405000.2020.1773199.

[fsn370335-bib-0052] Pedrali, D. , A. Scarafoni , A. Giorgi , and V. Lavelli . 2023. “Binary Alginate‐Whey Protein Hydrogels for Antioxidant Encapsulation.” Antioxidants 12: 1192. 10.3390/antiox12061192. 37371922 PMC10295361

[fsn370335-bib-0053] Pianet, I. , Y. André , M. A. Ducasse , et al. 2008. “Modeling Procyanidin Self‐Association Processes and Understanding Their Micellar Organization: A Study by Diffusion NMR and Molecular Mechanics.” Langmuir 24: 11027–11035. 10.1021/la8015904. 18767820

[fsn370335-bib-0054] Poklar Ulrih, N. 2017. “Analytical Techniques for the Study of Polyphenol–Protein Interactions.” Critical Reviews in Food Science and Nutrition 57: 2144–2161. 10.1080/10408398.2015.1052040. 26566184

[fsn370335-bib-0055] Popović, D. A. , D. D. Milinčić , M. B. Pešić , A. M. Kalušević , Ž. L. Tešić , and V. A. Nedović . 2019. “Encapsulation Technologies for Polyphenol‐Loaded Microparticles in Food Industry.” In Green Food Processing Techniques: Preservation, Transformation and Extraction, 335–367. Academic Press. 10.1016/b978-0-12-815353-6.00012-4.

[fsn370335-bib-0056] Rahmanian, N. , S. M. Jafari , and T. A. Wani . 2015. “Bioactive Profile, Dehydration, Extraction and Application of the Bioactive Components of Olive Leaves.” Trends in Food Science and Technology 42: 150–172. 10.1016/j.tifs.2014.12.009.

[fsn370335-bib-0057] Ramakrishna, S. , K. Fujihara , W. E. Teo , T. C. Lim , and Z. Ma . 2005. An Introduction to Electrospinning and Nanofibers, 396. World scientific. 10.1142/5894.

[fsn370335-bib-0058] Ranjbar‐Mohammadi, M. , and S. H. Bahrami . 2016. “Electrospun Curcumin Loaded Poly(ε‐Caprolactone)/gum Tragacanth Nanofibers for Biomedical Application.” International Journal of Biological Macromolecules 84: 448–456. 10.1016/j.ijbiomac.2015.12.024. 26706845

[fsn370335-bib-0059] Ranjbar‐Mohammadi, M. , M. Zamani , M. P. Prabhakaran , S. H. Bahrami , and S. Ramakrishna . 2016. “Electrospinning of PLGA/Gum Tragacanth Nanofibers Containing Tetracycline Hydrochloride for Periodontal Regeneration.” Materials Science and Engineering C 58: 521–531. 10.1016/j.msec.2015.08.066. 26478340

[fsn370335-bib-0060] Rezaei, A. , and A. Nasirpour . 2019. “Evaluation of Release Kinetics and Mechanisms of Curcumin and Curcumin‐β‐Cyclodextrin Inclusion Complex Incorporated in Electrospun Almond Gum/PVA Nanofibers in Simulated Saliva and Simulated Gastrointestinal Conditions.” BioNanoScience 9: 438–445. 10.1007/s12668-019-00620-4.

[fsn370335-bib-0061] Rezaei, A. , H. Tavanai , and A. Nasirpour . 2016. “Fabrication of Electrospun Almond Gum/PVA Nanofibers as a Thermostable Delivery System for Vanillin.” International Journal of Biological Macromolecules 91: 536–543. 10.1016/j.ijbiomac.2016.06.005. 27267574

[fsn370335-bib-0062] Rezaei, B. , A. M. Shoushtari , M. Rabiee , L. Uzun , A. P. F. Turner , and W. Cheung Mak . 2018. “Multifactorial Modeling and Optimization of Solution and Electrospinning Parameters to Generate Superfine Polystyrene Nanofibers.” Advances in Polymer Technology 37: 2743–2755. 10.1002/adv.21947.

[fsn370335-bib-0063] Rezaeinia, H. , B. Emadzadeh , and B. Ghorani . 2020. “Electrospun Balangu (Lallemantia Royleana) Hydrocolloid Nanofiber Mat as a Fast‐Dissolving Carrier for Bergamot Essential Oil.” Food Hydrocolloids 100: 105312. 10.1016/j.foodhyd.2019.105312.

[fsn370335-bib-0064] Sahoo, N. , R. K. Sahoo , N. Biswas , A. Guha , and K. Kuotsu . 2015. “Recent Advancement of Gelatin Nanoparticles in Drug and Vaccine Delivery.” International Journal of Biological Macromolecules 81: 317–331. 10.1016/j.ijbiomac.2015.08.006. 26277745

[fsn370335-bib-0065] Sanatkar, R. , G. Rahimi Kalateh Shah Mohammad , E. Karimi , E. Oskoueian , and R. Hendra . 2022. “Evaluation of Daidzein‐Loaded Chitosan Microcapsules for the Colon Cancer Drug Delivery: Synthesis, Characterization and Release Behaviour.” Polymer Bulletin 79, no. 9: 7391–7405. 10.1007/s00289-021-03853-0.

[fsn370335-bib-0066] Sathisaran, I. , and M. Balasubramanian . 2020. “Physical Characterization of Chitosan/Gelatin‐Alginate Composite Beads for Controlled Release of Urea.” Heliyon 6: e05495. 10.1016/j.heliyon.2020.e05495. 33251361 PMC7677684

[fsn370335-bib-0067] Schiffman, J. D. , and C. L. Schauer . 2008. “A Review: Electrospinning of Biopolymer Nanofibers and Their Applications.” Polymer Reviews 48: 317–352. 10.1080/15583720802022182.

[fsn370335-bib-0068] Serio, F. , A. F. Da Cruz , A. Chandra , et al. 2021. “Electrospun Polyvinyl‐Alcohol/Gum Arabic Nanofibers: Biomimetic Platform for In Vitro Cell Growth and Cancer Nanomedicine Delivery.” International Journal of Biological Macromolecules 188: 764–773. 10.1016/j.ijbiomac.2021.08.069. 34400233

[fsn370335-bib-0069] Shekarforoush, E. , F. Ajalloueian , G. Zeng , A. C. Mendes , and I. S. Chronakis . 2018. “Electrospun Xanthan Gum‐Chitosan Nanofibers as Delivery Carrier of Hydrophobic Bioactives.” Materials Letters 228: 322–326. 10.1016/j.matlet.2018.06.033.

[fsn370335-bib-0070] Siepmann, J. , and A. Göpferich . 2001. “Mathematical Modeling of Bioerodible, Polymeric Drug Delivery Systems.” Advanced Drug Delivery Reviews 48: 229–247. 10.1016/s0169-409x(01)00116-8. 11369084

[fsn370335-bib-0071] Silvestri, D. , J. Mikšíček , S. Wacławek , R. Torres‐Mendieta , V. V. T. Padil , and M. Černík . 2019. “Production of Electrospun Nanofibers Based on Graphene Oxide/Gum Arabic.” International Journal of Biological Macromolecules 124: 396–402. 10.1016/j.ijbiomac.2018.11.243. 30500492

[fsn370335-bib-0072] Soleimanifar, M. , S. M. Jafari , and E. Assadpour . 2020. “Encapsulation of Olive Leaf Phenolics Within Electrosprayed Whey Protein Nanoparticles; Production and Characterization.” Food Hydrocolloids 101: 105572. 10.1016/j.foodhyd.2019.105572.

[fsn370335-bib-0073] Stijnman, A. , I. Bodnar , and R. H. Tromp . 2011. “Electrospinning of Food‐Grade Polysaccharides.” Food Hydrocolloids 25, no. 5: 1393–1398. 10.1016/j.foodhyd.2011.01.005.

[fsn370335-bib-0074] Tavassoli‐Kafrani, E. , S. A. H. Goli , and M. Fathi . 2018. “Encapsulation of Orange Essential Oil Using Cross‐Linked Electrospun Gelatin Nanofibers.” Food and Bioprocess Technology 11: 427–434. 10.1007/s11947-017-2026-9.

[fsn370335-bib-0075] Teilaghi, S. , J. Movaffagh , and Z. Bayat . 2020. “Preparation as Well as Evaluation of the Nanofiber Membrane Loaded With *Nigella sativa* Extract Using the Electrospinning Method.” Journal of Polymers and the Environment 28: 1614–1625. 10.1007/s10924-020-01700-3.

[fsn370335-bib-0076] Teo, W. , and S. Ramakrishna . 2006. “A Review on Electrospinning Design and Nanofibre Assemblies.” Iopscience.Iop.Org 17: 89–106. 10.1088/0957-4484/17/14/r01. 19661572

[fsn370335-bib-0077] Torkamani, A. E. , Z. A. Syahariza , M. H. Norziah , A. K. M. Wan , and P. Juliano . 2018. “Encapsulation of Polyphenolic Antioxidants Obtained From *Momordica charantia* Fruit Within Zein/Gelatin Shell Core Fibers via Coaxial Electrospinning.” Food Bioscience 21: 60–71. 10.1016/j.fbio.2017.12.001.

[fsn370335-bib-0078] Vafania, B. , M. Fathi , and S. Soleimanian‐Zad . 2019. “Nanoencapsulation of Thyme Essential Oil in Chitosan‐Gelatin Nanofibers by Nozzle‐Less Electrospinning and Their Application to Reduce Nitrite in Sausages.” Food and Bioproducts Processing 116: 240–248. 10.1016/j.fbp.2019.06.001.

[fsn370335-bib-0079] Vardhanabhuti, B. , and S. Ikeda . 2006. “Isolation and Characterization of Hydrocolloids From Monoi ( *Cissampelos pareira* ) Leaves.” Food Hydrocolloids 20: 885–891. 10.1016/j.foodhyd.2005.09.002.

[fsn370335-bib-0080] Wang, P. , Y. Li , C. Zhang , F. Feng , and H. Zhang . 2020. “Sequential Electrospinning of Multilayer Ethylcellulose/Gelatin/Ethylcellulose Nanofibrous Film for Sustained Release of Curcumin.” Food Chemistry 308: 125599. 10.1016/j.foodchem.2019.125599. 31648098

[fsn370335-bib-0082] Wongsasulak, S. , M. Patapeejumruswong , J. Weiss , P. Supaphol , and T. Yoovidhya . 2010. “Electrospinning of Food‐Grade Nanofibers From Cellulose Acetate and Egg Albumen Blends.” Journal of Food Engineering 98: 370–376. 10.1016/j.jfoodeng.2010.01.014.

[fsn370335-bib-0083] Wulansari, R. , J. R. Mitchell , J. M. V. Blanshard , and J. L. Paterson . 1998. “Why Are Gelatin Solutions Newtonian?” Food Hydrocolloids 12: 245–249. 10.1016/s0268-005x(98)00038-1.

[fsn370335-bib-0084] Xu, L. Q. , K. G. Neoh , and E. T. Kang . 2018. “Natural Polyphenols as Versatile Platforms for Material Engineering and Surface Functionalization.” Progress in Polymer Science 87: 165–196. 10.1016/j.progpolymsci.2018.08.005.

[fsn370335-bib-0085] Yang, Z. , S. Chaieb , and Y. Hemar . 2021. “Gelatin‐Based Nanocomposites: A Review.” Polymer Reviews 61: 765–813. 10.1080/15583724.2021.1897995.

[fsn370335-bib-0086] Zhang, C. , and H. Zhang . 2018. “Formation and Stability of Core‐Shell Nanofibers by Electrospinning of Gel‐Like Corn Oil‐In‐Water Emulsions Stabilized by Gelatin.” Journal of Agricultural and Food Chemistry 66: 11681–11690. 10.1021/acs.jafc.8b04270. 30296080

[fsn370335-bib-0087] Zhang, R. , Z. Zhang , L. Zou , et al. 2016. “Enhancement of Carotenoid Bioaccessibility From Carrots Using Excipient Emulsions: Influence of Particle Size of Digestible Lipid Droplets.” Food & Function 7: 93–103. 10.1039/c5fo01172h. 26583923

